# Evaluation of novel epibatidine analogs in the rat nicotine drug discrimination assay and in the rat chronic constriction injury neuropathic pain model

**DOI:** 10.3389/adar.2023.11622

**Published:** 2023-09-11

**Authors:** Kevin Luque-Sanchez, Jasmine Felix, Joshua Bilbrey, Luis Restrepo, Morgan Reeves, Lance R. McMahon, Jenny L. Wilkerson

**Affiliations:** ^1^ Department of Pharmacodynamics, College of Pharmacy, University of Florida, Gainesville, FL, United States; ^2^ Department of Pharmaceutical Sciences, Texas Tech University Health Sciences Center, Amarillo, TX, United States

**Keywords:** neuropathic pain, drug abuse, nicotine, drug addiction, CCI-chronic constriction injury

## Abstract

Nicotine is the primary psychoactive component responsible for maintaining tobacco dependence in humans. Chronic pain is often a consequence of tobacco-related pathologies, and the development of a dual therapeutic that could treat chronic pain and tobacco dependence would be advantageous. Epibatidine reliably substitutes for nicotine in the drug discrimination assay, and is a potent analgesic, but has a side-effect profile that limits its therapeutic potential. Thus, considerable efforts to produce epibatidine derivatives are underway. Here we tested three epibatidine derivatives, 2′-fluoro-3'-(4-nitrophenyl)deschloroepibatidine (RTI-7527-102; i.e., RTI-102), 2′-fluorodeschloroepibatidine (RTI-7527-36; i.e., RTI-36), and 3'-(3″-dimethylaminophenyl)-epibatidine (RTI-7527-76; i.e., RTI-76) in both the rat nicotine drug discrimination assay as well as in the rat chronic constriction injury (CCI) of the sciatic nerve neuropathic pain model. Male and female Sprague-Dawley rats were trained on a fixed-ratio 10 schedule to discriminate nicotine (0.32 mg/kg base) from vehicle. All compounds dose-dependently substituted for nicotine, without significant decreases in response rates. In the discrimination assay the rank order potency was RTI-36 > nicotine > RTI-102 > RTI-76. Evidence suggests the α4β2* subtype is particularly important to nicotine-related abuse potential. Thus, here we utilized the antagonist dihydro-β-erythroidine (DHβE) to examine relative β2 subunit contribution. DHβE (3.2 mg/kg, s.c.) antagonized the discriminative stimulus effects of nicotine. However, relative to antagonism of nicotine, DHβE produced less antagonism of RTI-102 and RTI-76 and greater antagonism of RTI-36. It is likely that at nicotinic receptor subunits RTI-102, RTI-76 and RTI-36 possess differing activity. To confirm that the full discriminative stimulus of these compounds was due to nAChR activity beyond the β2 subunit, we examined these compounds in the presence of the non-selective nicotinic receptor antagonist mecamylamine. Mecamylamine (0.56 mg/kg, s.c.) pretreatment abolished nicotine-paired lever responding for all compounds. In a separate cohort, male and female Sprague-Dawley rats underwent CCI surgery and tested for CCI-induced mechanical allodynia via the von Frey assay. Each compound produced CCI-induced mechanical allodynia reversal. RTI-36 displayed higher potency than either RTI-102 or RTI-76. These novel epibatidine analogs may prove to be useful tools in the fight against nicotine dependence as well as novel neuropathic pain analgesics.

## Introduction

The nicotinic acetylcholine receptor (nAChR), specifically nAChR containing α4β2* subunits, have been widely studied for their critical role in nicotine dependence and addiction [1]. Efforts surrounding α4β2 nAChR have yielded the development of Chantix (varenicline), an α4β2 nAChR* partial agonist, as a Food and Drug Administration-approved smoking cessation therapeutic. Although varenicline is a useful smoking cessation pharmacotherapy, the largest individual cause of preventable death continues to be cigarette smoking [1, 2]. Thus, new therapeutics to treat nicotine addiction are desperately needed, and developing compounds that target α4β2* nAChR remains a viable option for developing novel smoking cessation therapeutics [1, 3–5]. Drug discrimination has utility in elucidating receptor mechanisms, and training nicotine as a discriminative stimulus allows for both the interrogation of novel compounds for activity at α4β2* nAChR as well as the ability to identify additional nAChR subunits or additional receptors that mediate the *in vivo* effects of nicotine [1, 3–5]. Specifically, if a compound is able to substitute for nicotine, then there is a good likelihood that the compound shares similar receptor pharmacology as nicotine, and likely α4β2* nAChR agonist activity.

Up to 17% of the US population live with neuropathic pain, which is associated with quality of life impairments and produced from injury to the nervous system [6–8]. Many conditions that produce neuropathic pain are increasingly prevalent in tobacco-dependent individuals as a consequence of long-term tobacco use and include chemotherapy induced peripheral neuropathy and peripheral diabetic neuropathy [2, 7]. A hallmark neuropathic pain symptom is mechanical allodynia, or light touch mechanical sensitivity [6–8]. Neuropathic pain is often poorly treated by tricyclic antidepressants, serotonin and noradrenaline reuptake inhibitors, antiepileptic drugs, and opioids. Unfortunately, these treatments provide only temporary relief, are susceptible to breakthrough pain, and have significant side effect profiles [8–11].

Many nAChR agonists, including those that act on α4β2* and α7 produce analgesia [3, 12]. However, the potential of α4β2* nAChR agonists as a viable chronic neuropathic pain therapeutic has yet to be realized. Epibatidine is classified as an α4β2 nAChR agonist, and initially much interest surrounded this compound as both an analgesic as well as a treatment for nicotine dependence, but a low therapeutic window due to toxicity led to its therapeutic abandonment [13–15].

Epibatidine derivatives including 2′-fluoro-3'-(4-nitrophenyl)deschloroepibatidine (RTI-7527-102; i.e., RTI-102), 2′-fluorodeschloroepibatidine (RTI-7527-36; i.e., RTI-36), and 3'-(3″-dimethylaminophenyl)-epibatidine (RTI-7527-76; I, e., RTI-76) have been developed to selectively target α4β2* nAChR activity. *In vitro* each compound has been characterized to have α4β2* nAChR binding affinity, with a reported Ki value of ∼0.037 for RTI-36, ∼0.009 for RTI-76, and ∼0.009 for RTI-102 [16–19]. These compounds possess differing nAChR subtype affinities from one another, in addition to α4β2* nAChR activity, and have a longer duration of action than epibatidine. Previous work in mice and in transfected frog oocytes demonstrated RTI-36 has the highest *in vivo* agonist potency out of the three RTI compounds at α4β2* nAChR and has agonist activity at α7 nAChR [4, 19]. RTI-76 has been found to exhibit partial α4β2* nAChR agonist activity, as well as a functional α2 nAChR agonist [4, 19]. RTI-102 has been found to act as an α4β2* nAChR antagonist *in vitro*, and a α4β2* nAChR partial agonist *in vivo* [4, 19]. This difference in activity at α4β2* nAChR may be due to species differences between the *in vitro* studies, which used transfected human α4β2* nAChR and *in vivo* studies, which have previously used mice [4, 19]. Here we sought to examine the pharmacology of these compounds in the rat drug discrimination assay. One cohort of male and female Sprague-Dawley rats were trained on a fixed-ratio 10 schedule to discriminate nicotine (0.32 mg/kg base) from vehicle, and varenicline, RTI-102, RTI-36 and RTI-76 were substituted for the nicotine discriminative stimulus. The nAChR β2 subunit is critical for mediating the reinforcing effects of nicotine [20]. Thus, we also examined the relative contribution that the β2 subunit plays in the nicotine discriminative stimulus substitution effects obtained with each of the epibatidine derivatives via the use of the β2* nAChR antagonist, dihydro-β-erythroidine (DHβE). To confirm nAChR agonist activity we also utilized the non-selective nAChR antagonist mecamylamine.

We next examined the analgesic effects of these novel epibatidine derivatives in a separate rat cohort that had undergone the chronic constriction injury (CCI) of the sciatic nerve neuropathic pain model and were tested for CCI-induced mechanical allodynia via the von Frey test [8]. The variety of nAChR subtype activity produced by these epibatidine derivatives, in addition to α4β2*, provides a plethora of drug development targets.

## Materials and methods

### Animals

Drug discrimination studies: Adult female and male Sprague Dawley rats (Taconic Biosciences, Germantown, NY, *N* = 4 per sex, *N* = 8 total), weighing approximately 250 g and 300 g upon arrival, respectively, were housed individually in a temperature- (21.9°C ± 1.9°C) and humidity-controlled (53% ± 14%) vivarium with a 12-h light/dark cycle. After a brief acclimation period, individual body weights were maintained at no less than 85% of free feeding body weight as well as no less than 2.5 of Body Conditioning Score, by adjusting daily food rations. Access to chow (Dustless Precision Pellets Grain-Based Rodent Diet, Bio-Serv, Frenchtown, NJ) was provided in the rats’ home cages approximately 30 min following daily experimental sessions. In addition to chow consumption, rats consumed a maximum of fifty 45-mg sucrose pellets (Dustless Precision Pellets^®^ 45 mg, Sucrose, Bio-Serv) available during experimental sessions for the drug discrimination assay as described below.

Neuropathic pain studies: Adult female and male Sprague Dawley rats (Taconic Biosciences, Germantown, NY, *N* = 3 per sex, *N* = 6 total), weighing approximately 250 g and 300 g upon arrival, respectively, were pair-housed in a temperature- (21.9°C ± 1.9°C) and humidity-controlled (53% ± 14%) vivarium with a 12-h light/dark cycle (lights on at Eastern Daylight Time 07:00 h) during which food (2,918 Teklad global 18% protein rodent diets, Envigo, Frenchtown, NJ) and reverse osmosis-purified water were available at all times. The animal protocol was approved by the Institutional Animal Care and Use Committee (IACUC) at the University of Florida, accredited by the Association for Assessment and Accreditation of Laboratory Animal Care International (AAALAC) and in accordance with the National Institutes of Health (NIH) Guide for the Care and Use of Laboratory Animals.

### Drugs

(−)-Nicotine hydrogen tartrate salt was obtained from Sigma Chemical (St. Louis, MO). Varenicline dihydrochloride was obtained from the National Institute on Drug Abuse drug supply program (Rockville, MD). Mecamylamine was obtained from Waterstone Technology (Camel, IN). The epibatidine derivatives 2′-Fluorodeschloroepibatidine (RTI-7527-36), 3′-(3″-dimethylaminophenyl) epibatidine (RTI-7527-76), and 2′-fluoro-3′-(4-nitrophenyl) deschloroepibatidine (RTI-7527-102) were synthesized at the Center for Organic and Medicinal Chemistry, Research Triangle Institute (Research Triangle Park, NC) as previously described [16–18]. Dihydro-β-erythroidine hydrobromide was obtained from Tocris (Minneapolis, MN). Drugs were dissolved in 0.9% physiological saline and administered s.c. (except mecamylamine which was administered intraperitoneally) in a volume of 10 mL/kg. The dose of nicotine is expressed as the weight of the free base. Other drug doses are expressed as the weight of the base plus the salt.

### Drug discrimination

The equipment, procedures, and animal training techniques utilized in the drug discrimination assay were as previously described [21] and are fully described within the Supplementary Methods section. In brief, eight operant-conditioning chambers (Model ENV-203; Med Associates Inc., Fairfax, VT) measuring 25-cm long × 25-cm wide × 31-cm high per chamber, enclosed within a sound-attenuating cubicle were used for these experiments. For the drug discrimination experiments Med-PC software version V (Med Associates Inc., Fairfax, VT) was used to control experimental events and provided an electronic version of data generated. Rats underwent lever-response shaping as previously described [21]. Each rat was placed daily in its assigned chamber each day during the light period for seven consecutive days/week. Each rat was returned to their home cage at the end of a session, and 30 min later was fed a ration of food to maintain body weight. Throughout training sessions, a single lever press was sufficient to turn off LED lights and to activate the pellet dispenser for 0.1 s [fixed-ratio (FR) 1 schedule]. After pellet dispenser activation, a 0.1-s time-out period occurred where LEDs turned off, the house light turned on, and lever responses did not produce an effect. During this time-out period, the retractable levers remained out. The levers were counterbalanced, and the right-versus left-assignment of lever presentation was switched daily (i.e., right–left–right–left). After a rat received the maximum 50 food pellets per session during four consecutive sessions under a FR1 and FR3 schedule of reinforcement and for two consecutive sessions under the FR5 schedule of reinforcement, the fixed ratio was increased to FR10. After a rat received 50 food pellets per session under a FR10 for two consecutive training sessions, the rat was eligible for drug discrimination training.

#### Drug discrimination training

Experimental subjects were placed into a nicotine (0.32 mg/kg, s.c., administered 30 min prior to sessions) training group and underwent discriminative stimulus training as previously described [21], and fully described within the Supplementary Methods. In brief, following an injection of either the training dose or vehicle, subjects were returned to their home cage, and then placed into their assigned chamber after a 30-min pretreatment. During the training session each downward lever press activated the pellet dispenser under a FR10 schedule of reinforcement. The left-versus right lever assignment was switched daily (i.e., left–right–left–right). Test sessions commenced when the following criteria were met individually for at least four consecutive sessions under the FR10-response schedule of reinforcement: 1) Out of the total responses at least 80% were correct and 2) Prior to the delivery of the first reinforcer less than ten incorrect responses were made. After the first test session, each subject repeatedly underwent test sessions each time when the test criteria were met for at least one drug- and at least one vehicle-appropriate responding under the FR10-response schedule of reinforcement. Rats acquired the discrimination of 0.32 mg/kg nicotine base from saline in a mean of 68 sessions (range 58–76) including both nicotine and saline training sessions.

#### Testing

Test sessions were identical to the training sessions, except ten responses on either lever resulted in delivery of food and doses of test compounds were administered, as previously described [21]. Dose-effect assessments of each training drug were obtained once at the beginning of the study and a second time after tests with all other drugs were completed. Antagonists were administered 15 min before the test compounds. Over 1.5 years, approximately two drug tests were completed each week.

### Behavioral assessment of allodynia

Rats were assessed for behavioral responses in the von Frey assay as previously described [8]. Briefly, the von Frey testing area was comprised of bars that were 2-mm-thick, parallel, and spaced 8 mm apart. Rats were placed on top of the bars and were habituated to the von Frey testing area approximately 45 min for 5 days. In a sound-, light-, and temperature-controlled room, testing occurred during the first half of the light cycle. The von Frey test utilizes a series of calibrated monofilaments (3.61–5.18 log stimulus intensity; North Coast Medical, Morgan Hills, CA, United States) applied randomly to the left and right plantar surface of the hind paw for 8 s. Shaking, licking, or lifting the paw was considered a response. Baseline responses in the von Frey assay were determined prior to surgery. Experimenter testing was conducted blinded to surgical and drug treatment.

### Chronic constriction injury (CCI) surgery

Following baseline von Frey assessment, CCI or sham surgeries were completed as previously described [8]. Briefly, in isoflurane (induction 5% vol. followed by 2.5% in oxygen)-anesthetized rats, the dorsal left thigh was shaved. The sciatic nerve was identified via aseptic procedures, isolated, and ligated loosely using a total of 4 chromic gut suture segments (Ethicon, Somerville, NJ, United States). All procedures during the sham surgery were identical to those procedures for the CCI surgery except the nerve was not ligated. The overlying muscle overlying the sciatic nerve was closed via [2] 3–0 sterile silk sutures (Ethicon). Within approximately 5 min all animals recovered from anesthesia. Placement into surgical CCI or sham groups was randomly assigned.

### Data analysis

Data from the drug discrimination assay are shown as an overall percent of the drug-appropriate response achieved, out of total response numbers [21]. Response rate is expressed as the percentage of the control response rate for each eat, defined as the mean response rate from the five saline training sessions immediately preceding the test [21]. In all assays, when a compound produced a mean effect greater than 50%, the effective dose (ED)_50_ values and corresponding 95% confidence limits were calculated using linear regression [21]. However, if a compound failed to produce a 50% or greater effect, an ED_50_ value was not calculated. Potency ratios and 95% confidence limits (CL) were calculated as the ratio of ED_50_ values calculated from the dose-response curves [21 22]. If a potency ratio did not include 1 within the 95% CL, this indicated a statistically significant difference in potency. Data from dose-response experiments were analyzed via one-way repeated measures analysis of variance (ANOVA). A Bonferroni comparison was used for *post hoc* analysis following a significant main effect and/or interactive effect (*p* < 0.05). GraphPad Prism version 6.0 (GraphPad Software Inc., San Diego, CA, United States) was used for analyses. For all experiments, the mean ± SEM is used to express data.

## Results

### Nicotine drug discrimination and substitution tests

**FIGURE 1:**
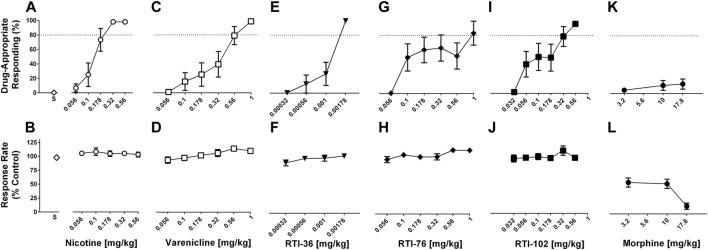
Discriminative stimulus (top row) and rate-decreasing (bottom row) effects in rats trained to discriminate 0.32 mg/kg nicotine from saline. **(A)** Nicotine dose-relatedly produces a nicotine (0.32 mg/kg) discriminative stimulus, and **(B)** rate-decreasing effects. **(C)** Varenicline, **(E)** RTI-36, **(G)** RTI-76, and **(I)** RTI-102 all dose-relatedly substitute for the nicotine discriminative stimulus and **(D, F, H, J)** respective rate-decreasing effects. **(K)** Discriminative stimulus and **(L)** rate-decreasing effects of morphine. Top panels show drug-appropriate responding on the ordinates and drug dose in mg/kg (log scale) on the abscissae. The dashed line represents the percent responding on the nicotine-paired lever required to meet full discriminative stimulus criteria. Bottom panels show response rate normalized to saline control on the ordinates as function of drug dose in mg/kg (log scale) on the abscissae. Data reflect mean ± SEM, *n* = 8 rats.

All drug discrimination statistical analysis data is shown in Table 1; Supplementary Table S1. The first determination of the nicotine dose response function was not significantly different from the nicotine dose-response determination conducted at the end of experiments (*p* > 0.05) (Supplementary Figure S1; Supplementary Table S1). Nicotine dose-dependently increased the percentage of responses on the nicotine-appropriate lever to 98% whereas saline produced 0.4% nicotine-appropriate responses (Figure 1A). The ED_50_ value (95% confidence limits) of nicotine to produce discriminative stimulus effects was 0.12 (0.09–0.17) mg/kg (Table 1). Nicotine did not decrease the response rate at any dose tested (Figure 1B). Varenicline (Figures 1C, D), RTI-36 (Figures 1E, F), RTI-76 (Figures 1G, H), and RTI-102 (Figures 1I, J) dose dependently increased nicotine-appropriate responding to a maximum of 99% at 1 mg/kg varenicline, 99% at 0.00178 mg/kg RTI-36, 83% at 1 mg/kg RTI-76, and 96% at 0.56 mg/kg RTI-102. The ED_50_ values for nicotine-like discriminative stimulus effects were 0.30 mg/kg for varenicline, 0.001 mg/kg for RTI- 36, 0.2 mg/kg for RTI- 76, and 0.12 mg/kg for RTI-102 (Table 1). The maximum percentage of nicotine appropriate responding produced by morphine was 13% (Figures 1K, L); an ED_50_ value was not calculated for this drug.

**TABLE 1:** Drug Discrimination statistics.

	Alone		3.2 mg/kg DHβE pretreatment			0.56 mg/kg mecamylamine pretreatment	
	Statistical Analysis	ED_50_ (95% Confidence limits) mg/kg	Statistical Analysis	ED_50_ (95% Confidence limits) mg/kg	Potency Ratio (DHβE/Alone)	Statistical Analysis	ED_50_ (95% Confidence limits) mg/kg
Nicotine	F (7, 28) = 17.96 *p* < 0.0001	0.12 (0.09–0.17)	F (7, 14) = 24.30 *p* < 0.01	0.55 (0.47–0.63)	4.58 (3.70–5.22)	F (6, 25) = 1.115 *p* = 0.3622	N.A.
Varenicline	F (7, 38) = 11.49 *p* < 0.001	0.30 (0.21–0.42)	F (7, 42) = 16.60 *p* < 0.001	0.49 (0.38–0.63)	1.63 (0.90–3)	N.A.	N.A.
RTI-102	F (7, 35) = 10.57 *p* < 0.001	0.12 (0.09–0.17)	F (6, 24) = 5.970 *p* < 0.05	0.25 (0.19–0.34)	2.08 (2.00–2.11)	*p* = 0.4595	N.A.
RTI-76	F (7, 36) = 3.792 *p* < 0.05	0.20 (0.15–0.29)	F (6, 12) = 9.133 *p* < 0.05	0.41 (0.26–0.64)	2.05 (1.73–2.21)	F (6, 12) = 2.543 *p* = 0.1598	N.A.
RTI-36	F (7, 21) = 24.85 *p* < 0.0001	0.001 (0.0008–0.0012)	F (6, 18) = 5.387 *p* < 0.05	0.009 (0.003–0.031)	9.00 (3.75–25.83)	F (6, 18) = 0.7133 *p* = 0.4405	N.A.
Morphine	F (7, 14) = 0.6083 *p* = 0.5558	N.A.	N.A.	N.A.	N.A.	N.A.	N.A.

### DHβE in combination with saline, nicotine, varenicline, RTI-36, RTI-76, RTI-102

**FIGURE 2:**
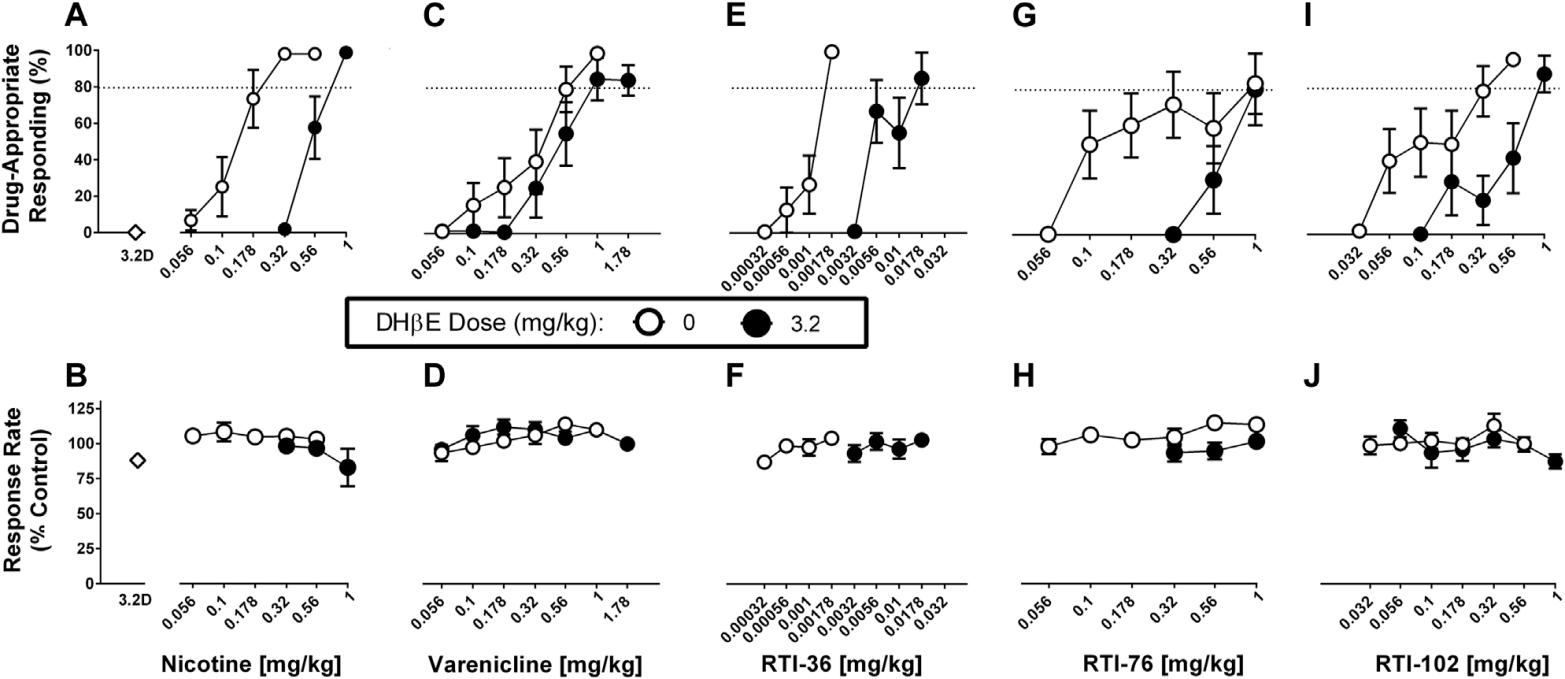
DHβE (3.2 mg/kg) antagonism of discriminative stimulus (top row) and rate-decreasing (bottom row) effects in rats trained to discriminate 0.32 mg/kg nicotine from saline. **(A)** DHβE produces a rightward shift of the nicotine dose response function for the nicotine (0.32 mg/kg) discriminative stimulus, and **(B)** response rate effects. **(C)** DHβE does not alter varenicline dose response function for the nicotine discriminative stimulus and **(D)** response rate effects. DHβE does produce a rightward shift of the **(E)** RTI-36, **(G)** RTI-76, and **(I)** RTI-102 dose response curve in the substitution effects of the nicotine discriminative stimulus and **(F, H, J)** respective rate-decreasing effects. Top panels show drug-appropriate responding on the ordinates and drug dose in mg/kg (log scale) on the abscissae. The dashed line represents the percent responding on the nicotine-paired lever required to meet full discriminative stimulus criteria. Bottom panels show response rate normalized to saline control on the ordinates as function of drug dose in mg/kg (log scale) on the abscissae. Data reflect mean ± SEM, *n* = 7–8 rats (one rat was lost to attrition).

DHβE (1, 3.2 mg/kg) in combination with saline produced no more than 1% drug-appropriate responding in the nicotine discrimination assay and neither dose significantly decreased response rates (Figure 2A; Supplementary Figure S2). The dose of 1 mg/kg DHβE did not significantly alter either nicotine or varenicline discrimination dose-response functions (Supplementary Figure S2; Supplementary Table S1). The dose of 3.2 mg/kg DHβE produced significant rightward shifts in the nicotine (Figures 2A, B), RTI-36 (Figures 2E, F), RTI-76 (Figures 2G, H) and RTI-102 (Figures 2I, J) discrimination dose-response functions (Table 1). The ED_50_ values for nicotine-like discriminative stimulus effects were 0.55 mg/kg for 3.2 mg/kg DHβE + nicotine, 0.009 mg/kg for 3.2 mg/kg DHβE + RTI-36, 0.41 mg/kg for 3.2 mg/kg DHβE + RTI-76, and 0.25 mg/kg for 3.2 mg/kg DHβE + RTI-102 (Table 1). Although the dose of 3.2 mg/kg DHβE produced a small rightward shift of the varenicline discriminative stimulus dose response curve (Figures 2C, D), with a resulting ED_50_ value of 0.49 mg/kg, this shift in potency was not significant as the potency ratio (95% confidence limits) was 1.63 (0.90–3) (Table 1). No dose combination of 3.2 mg/kg DHβE significantly altered response rates.

### Mecamylamine in combination with saline, nicotine, RTI-36, RTI-76, RTI-102

**FIGURE 3:**
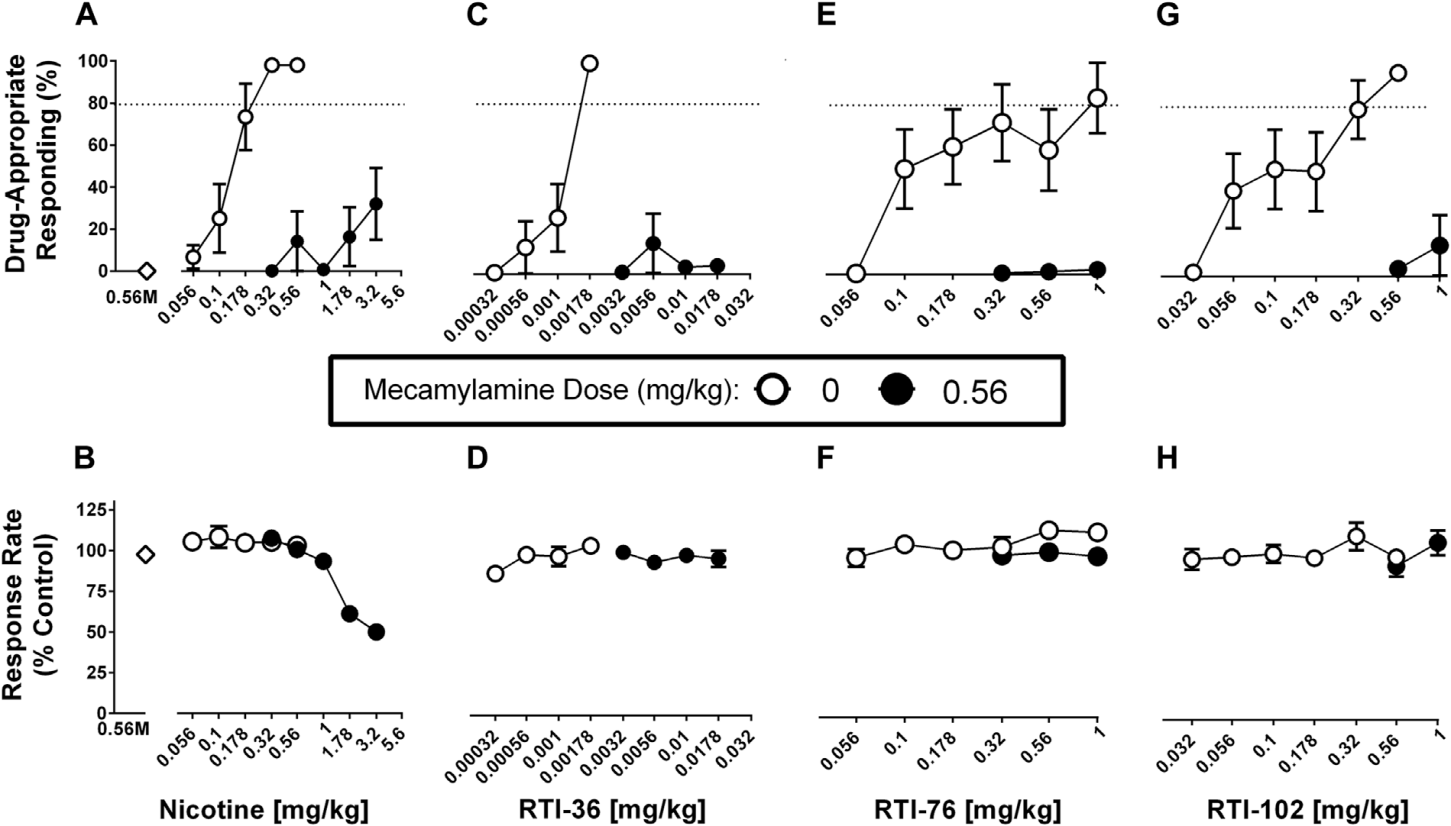
Mecamylamine (0.56 mg/kg) antagonism of discriminative stimulus (top row) and rate-decreasing (bottom row) effects in rats trained to discriminate 0.32 mg/kg nicotine from saline. **(A)** Mecamylamine abolishes the varenicline dose response function for the nicotine (0.32 mg/kg) discriminative stimulus, and **(B)** response rate effects. Mecamylamine abolishes the **(C)** RTI-36, **(E)** RTI-76, and **(G)** RTI-102 dose response curve in the substitution effects of the nicotine discriminative stimulus and **(D, F, H)** respective rate-decreasing effects. Top panels show drug-appropriate responding on the ordinates and drug dose in mg/kg (log scale) on the abscissae. The dashed line represents the percent responding on the nicotine-paired lever required to meet full discriminative stimulus criteria. Bottom panels show response rate normalized to saline control on the ordinates as function of drug dose in mg/kg (log scale) on the abscissae. Data reflect mean ± SEM, *n* = 7–8 rats (one rat was lost to attrition).

Mecamylamine (0.56 mg/kg) in combination with saline produced no more than .15% drug-appropriate responding in the nicotine discrimination assay and this dose did not significantly decrease response rates (Figures 3A, B). Mecamylamine (0.56 mg/kg) significantly antagonized nicotine (Figures 3A, B), RTI-36 (Figures 3C, D), RTI-76 (Figures 3E, F), and RTI-102 (Figures 3G, H) discrimination responding. Mecamylamine (0.56 mg/kg) + nicotine (3.2 mg/kg) produced 32% responding on the nicotine appropriate lever, and decreased response rates to 50% of control values (Table 1). The dose combinations of mecamylamine paired with 1.78 and 3.2 mg/kg nicotine were the only mecamylamine dose combinations that significantly altered response rates.

### Effects of RTI-102, RTI-36, and RTI 76 on CCI-induced mechanical allodynia

**FIGURE 4:**
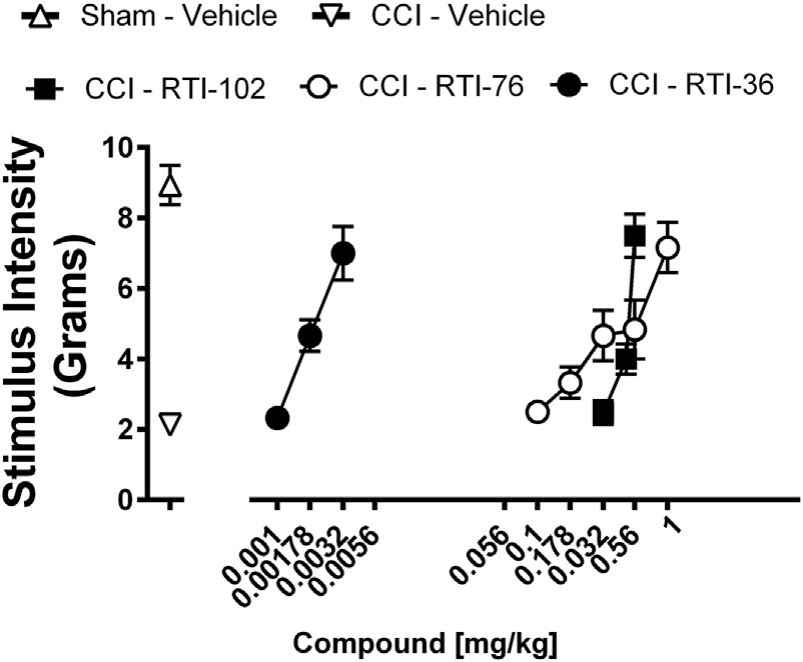
Epibatidine derivatives attenuate CCI-induced mechanical allodynia RTI-36, RTI-76, and RTI-102 dose-relatedly attenuate CCI-induced mechanical allodynia Abscissae: drug dose (mg/kg, *ip*), log scale; ordinates: the force in grams to produce a threshold response. Data reflect mean ± SEM, *n* = 6 rats.

Prior to surgery, all rats displayed similar baseline behavioral von Frey thresholds [*p* = 0.6631]. The response thresholds of sham rats (Figure 4) did not significantly differ from their pre-injection baseline [*p* = 0.9259]. However, CCI (Figure 4) produced mechanical allodynia as indicated by the decrease in stimulus intensity [*p* < 0.0001]. All three compounds, RTI-102, RTI-36, and RTI 76 dose dependently reversed CCI-induced allodynia (Figure 4; Table 2). The respective ED_50_ values (95% confidence limits) of RTI-102, RTI-36, and RTI 76 to reverse CCI-induced mechanical allodynia were 0.44 (0.41–0.48) mg/kg, 0.002 (0.0009–0.0028) mg/kg, and 0.25 (0.29–0.42) mg/kg (Table 2). The rank order potency of these compounds to reverse mechanical allodynia were RT1-36 > RTI-76 > RTI-102.

**TABLE 2:** Statistical analysis results for CCI-induced Mechanical Allodynia Experiments.

	Statistics	ED50 (95% confidence limits) mg/kg
RTI-102	F (2, 33) = 29.82 *p* < 0.0001	0.44 (0.41–0.48)
RTI-76	F (4, 55) = 7.952 *p* < 0.0001	0.35 (0.29–0.42)
RTI-36	F (2, 33) = 19.72 *p* < 0.0001	0.002 (0.0009–0.0028)

## Discussion

Here we show that RTI-36, RTI-102, and RTI-76 dose-dependently reversed CCI-induced mechanical allodynia. These three compounds, RTI-36, RTI-102, and RTI-76, also fully substituted for 0.32 mg/kg nicotine (0.32 mg/kg) in the nicotine drug discrimination assay. Varenicline, a commonly prescribed drug for nicotine addiction, produced full nicotine substitution. We examined the relative contribution of the β2 subunit with DHβE pretreatment. DHβE (3.2 mg/kg, s.c.) antagonized the discriminative stimulus effects of nicotine but not varenicline. However, relative to antagonism of nicotine, DHβE produced less antagonism of RTI-102 and RTI-76 and greater antagonism of RTI-36. To confirm that the full discriminative stimulus of these compounds was due to nAChR activity beyond the β2 subunit, we examined these compounds in the presence of the non-selective nicotinic receptor antagonist mecamylamine. Mecamylamine (0.56 mg/kg, s.c.) pretreatment abolished nicotine-paired lever responding for all compounds.

The nicotine discrimination assay was pharmacologically selective, as morphine only produced a ∼13% nicotine substitution. The ED_50_ values for nicotine-like discriminative stimulus effects were 0.12 mg/kg for nicotine, 0.001 mg/kg for RTI- 36, 0.12 mg/kg for RTI-102, and 0.2 mg/kg for RTI- 76, The relative potency of epibatidine derivates reported here is supportive of previous *in vitro* work demonstrating RTI-36 and RTI-102 have higher binding affinity at αβ-containing nAChR subtypes than RTI-76 [16–18]. In mice discriminating nicotine (1 mg/kg, s.c.) RTI-102 failed to fully substitute for the nicotine discriminative stimulus [4]. Here, in rats RTI-102 produced full substitution for a lower nicotine (0.32 mg/kg, s.c.) training dose. These observed differences in the substitution of RTI-102 for nicotine in the drug discrimination assay may be due to a species difference between rats and mice. Additionally, it is well established that the dose of the training drug influences the ability of various test compounds to substitute for the training drug, with higher training doses having greater discriminative stimulus receptor specificity. The rank order potency of nicotine and the epibatidine derivatives to be antagonized by DHβE was RTI-36 > nicotine > RTI-76 = RTI-102. The relative potency of epibatidine derivates reported here is supportive of previous *in vitro* work that demonstrated RTI-36 and RTI-102 have higher binding affinity at αβ-containing nAChR subtypes than RTI-76 [16–18], and that RTI-102 has lower efficacy at α4β2 nAChR than nicotine [23–25]. Differences in α4β2 nAChR efficacy may underlie the observed potency of each compound to substitute for nicotine discriminative stimulus effects. However, the failure of DHβE to antagonize the discriminative stimulus effects of varenicline, which fully substituted for the nicotine discriminative stimulus has similarly been demonstrated in mice trained to discriminate nicotine [4, 5, 26]. Thus, with regard to the *in vivo* activity of both RTI-102 and varenicline, these findings suggest an alternative conclusion. *In vitro*, both RTI-102 and varenicline have less efficacy at the α4β2 nAChR than nicotine [23–25]. DHβE is assumed to be a competitive β2 nAChR antagonist. Varenicline is a full agonist at the α7 subunit [27] and it appears that nAChRs containing the α5 subunit may mediate at least some of the physiological effects of varenicline [28, 29]. Therefore, other nAChR receptor subunits in addition to α4β2 subunits could have a contribution to the nicotine-related discriminative stimulus effects and the nAChR agonists used in this study, including both varenicline and RTI-102, which produced nicotine substitution [30].

The nonselective nAChR antagonist mecamylamine (0.56 mg/kg) attenuated the discriminative effects of nicotine, as well as the substitution effects of varenicline and the three epibatidine derivatives, reinforcing previous studies that conclude nicotine exerts discriminative stimulus effects through nAChRs. It should be noted that DHβE-induced antagonism of nicotine, RTI-36, RTI-102, and RTI-76 substitution in the nicotine drug discrimination assay was surmounted at drug doses that did not disrupt schedule-controlled responding. However, mecamylamine-induced antagonism of nicotine drug discrimination was not surmountable, at least regarding nicotine, up to doses (i.e., 1.78 and 3.2 mg/kg nicotine) that produced significant operant rate decreases. Likewise, mecamylamine-induced antagonism of RTI-36, RTI-102, and RTI-76 nicotine substitution was not surmountable at any dose of the epibatidine derivatives tested. Due to limited compound amounts, the doses of epibatidine derivatives were not studied to the observational point of operant rate decreases. Here we found that in the presence of mecamylamine, nicotine response rates were significantly decreased at higher drug doses (i.e., 1.78 and 3.2 mg/kg) with only partial nicotine stimulus discrimination, an effect not seen when nicotine was administered alone at partial stimulus discrimination doses (i.e., 0.01 and 0.0178 mg/kg). One interpretation of this finding is that at higher doses (i.e., 1.78 and 3.2 mg/kg) nicotine has activity at receptors other than nAChR [28]. Taken together, the current studies support the idea that each of the epibatidine derivatives possess differing nAChR subunit activity and differing α4β2 nAChR potency, which could produce useful analgesic profiles.

Nicotine-related analgesic effects have been established since 1932 [31]. Activation of nAChR in the spinal cord leads to the release of neurotransmitters believed to play a role in pain modulation, including acetylcholine, noradrenaline, serotonin, gamma-aminobutyric acid, and opioid peptides [32–34]. Due to the modest analgesic potency of nicotine and its substantial safety liabilities the use of nicotine as a clinical analgesic has not materialized [35]. In preclinical models nAChR agonists have shown activity in mitigating pain-related behaviors from numerous different etiologies, including acute nociceptive, inflammatory, and neuropathic pain [3, 12, 36]. Considerable issues regarding the development of nAChR analgesics generally include a relatively narrow therapeutic dosing window where beneficial effects are separated from toxic effects, as well as sex-related potency and efficacy differences [37]. Epibatidine is 200 times more potent than morphine to produce analgesic effects [38]. However, epibatidine is extremely toxic, causing hypertension, respiratory paralysis, and seizures, with death occurring at doses not much higher than those required for antinociception [39, 40].

Although not studied in this current report, in mice epibatidine produced significant hypothermia and nicotine substitution in mice trained to discriminate nicotine [4]. From this study, the ED_50_ for epibatidine to produce nicotine (1 mg/kg base, s.c.) substitution was 0.002 mg/kg, while the ED_50_ for epibatidine to produce hypothermia was 0.005 mg/kg [4]. This was compared to the doses of each epibatidine analog studied in the current report. Indeed, in mice, it was found that RTI-36, RTI-102, and RTI-76 produced significant hypothermia, with reported 0.07, 3.0 and 4.3 mg/kg ED_50_ values, respectively [4]. The reported ED_50_ values (in mg/kg) for these compounds to produce nicotine cross-substitution were 0.023, no substitution, and 2.29 for RTI-36, RTI-102, and RTI-76, respectively [4]. A limitation of the current study is that Sprague Dawley rats are less sensitive to nicotine-induced hypothermia than other rat strains (i.e., Flinders Sensitive Line rats), and hypothermia was not measured in the current study [41]. In future studies, the use of hypothermia sensitive rat strains would lead to a better characterization of therapeutic vs. hypothermic (i.e., toxic) effects. In previous mouse studies these epibatidine derivatives were found to display limited acute antinociceptive activity in mice subjected to the hot plate nociception assay [24].

Although epibatidine may be of little clinical use, epibatidine derivatives may be useful analgesics if they display analgesic-like effects at doses that do not display toxicity or other side effects. In our hands RTI-36, RTI-102, and RTI-76 produced full reversal of CCI-induced mechanical allodynia in both male and female rats. Although there were potency differences, doses that reversed mechanical allodynia did not produce lethality. However, each compound produced full mechanical allodynia reversal at doses that fully substituted for nicotine, and indeed each compound was more potent in the nicotine drug discrimination assay than the von Frey assay. As nicotine is highly addictive, the abuse and dependence potential of these compounds should be further explored before these derivatives are considered as viable potential novel analgesics. Taken together, these findings suggest more work on the receptor selectivity and binding activity of the scaffolds of these compounds may yield better therapeutic leads.

Here we show that each epibatidine derivative dose-dependently reversed CCI-induced mechanical allodynia and fully substituted for 0.32 mg/kg nicotine (0.32 mg/kg) in the nicotine drug discrimination assay. Activity in both assays was likely due to differences in potency at α4β2* nAChRs as well as effects on different nAChR subunits. In addition, the contribution of receptor activity beyond nAChRs cannot be ruled out. However, these studies support the idea that neuronal nAChR agonists could serve the dual purpose of a smoking cessation aid and analgesic. The utility of epibatidine derived compounds for both therapeutic indications could be realized if a compound lacks significant abuse and dependence liabilities as well as achieves a suitable therapeutic window.

## Data Availability

The datasets presented in this study can be found in online repositories. The names of the repository/repositories and accession number(s) can be found below: https://osf.io[DOI 10.17605/OSF.IO/PYGUA].

## Abbreviations

ANOVA, analysis of variance; CCI, chronic constriction injury; CL, confidence limits; DHβE, dihydro-β-erythroidine; ED, effective dose; FR, fixed ratio; nAChR, nicotinic acetylcholine receptor; s.c., subcutaneous.

## References

[1] Moerke MJ McMahon LR Wilkerson JL. More than smoke and patches: the quest for pharmacotherapies to treat tobacco use disorder. Pharmacol Rev (2020) 72(2):527–57. 10.1124/pr.119.018028 32205338 PMC7090325

[2] U.S. Department of Health and Human Services. The health consequences of smoking: 50 years of progress. Atlanta, GA: A Report of the Surgeon General (2014). U.S. department of health and human services, centers for disease control and prevention, national center for chronic disease prevention and health promotion, office on smoking and health.

[3] Wilkerson JL Deba F Crowley ML Hamouda AK McMahon LR. Advances in the *in vitro* and *in vivo* pharmacology of alpha4beta2 nicotinic receptor positive allosteric modulators. Neuropharmacology (2020) 168:108008. 10.1016/j.neuropharm.2020.108008 32113032 PMC7113117

[4] Rodriguez JS Cunningham CS Moura FB Ondachi P Carroll FI McMahon LR. Discriminative stimulus and hypothermic effects of some derivatives of the nAChR agonist epibatidine in mice. Psychopharmacology (Berl) (2014) 231(23):4455–66. 10.1007/s00213-014-3589-z 24800895 PMC4224623

[5] de Moura FB McMahon LR. The contribution of α4β2 and non-α4β2 nicotinic acetylcholine receptors to the discriminative stimulus effects of nicotine and varenicline in mice. Psychopharmacology (Berl) (2017) 234(5):781–92. 10.1007/s00213-016-4514-4 28028600 PMC5309148

[6] International Association for the Study of Pain. IASP taxonomy. Pain terms. Neuropathic pain (2017).

[7] Cavalli E Mammana S Nicoletti F Bramanti P Mazzon E. The neuropathic pain: an overview of the current treatment and future therapeutic approaches. Int J Immunopathol Pharmacol (2019) 33:2058738419838383. 10.1177/2058738419838383 30900486 PMC6431761

[8] Wilkerson JL Gentry KR Dengler EC Wallace JA Kerwin AA Armijo LM Intrathecal cannabilactone CB(2)R agonist, AM1710, controls pathological pain and restores basal cytokine levels. Pain (2012) 153(5):1091–106. 10.1016/j.pain.2012.02.015 22425445 PMC3603341

[9] Coupland C Hill T Morriss R Arthur A Moore M Hippisley-Cox J. Antidepressant use and risk of suicide and attempted suicide or self harm in people aged 20 to 64: cohort study using a primary care database. BMJ (2015) 350:h517. 10.1136/bmj.h517 25693810 PMC4353276

[10] Piccolo J Kolesar JM. Prevention and treatment of chemotherapy-induced peripheral neuropathy. Am J Health-System Pharm (2014) 71(1):19–25. 10.2146/ajhp130126 24352178

[11] Hylands-White N Duarte RV Raphael JH. An overview of treatment approaches for chronic pain management. Rheumatol Int (2017) 37(1):29–42. 10.1007/s00296-016-3481-8 27107994

[12] Bagdas D Gurun MS Flood P Papke RL Damaj MI. New insights on neuronal nicotinic acetylcholine receptors as targets for pain and inflammation: a focus on α7 nAChRs. Curr Neuropharmacol (2018) 16(4):415–25. 10.2174/1570159X15666170818102108 28820052 PMC6018191

[13] Bannon AW Decker MW Holladay MW Curzon P Donnelly-Roberts D Puttfarcken PS Broad-spectrum, non-opioid analgesic activity by selective modulation of neuronal nicotinic acetylcholine receptors. Science (1998) 279:77–81. 10.1126/science.279.5347.77 9417028

[14] Spande TF Garraffo HM Edwards MW Yeh HJ Pannell L Daly JW. Epibatidine: a novel (chloropyridyl) azabicycloheptane with potent analgesic activity from an ecuadoran poison frog. J Am Chem Soc (1992) 114:3475–8. 10.1021/ja00035a048

[15] Sullivan JP Decker MW Brioni JD Donnelly-Roberts D Anderson DJ Bannon AW (+/−)-Epibatidine elicits a diversity of *in vitro* and *in vivo* effects mediated by nicotinic acetylcholine receptors. J Pharmacol Exp Ther (1994) 271:624–31. 7965777

[16] Carroll FI Ma W Deng L Navarro HA Damaj MI Martin BR. Synthesis, nicotinic acetylcholine receptor binding, and antinociceptive properties of 3'-(substituted phenyl)epibatidine analogues. Nicotinic partial agonists. J Nat Prod (2010) 73(3):306–12. 10.1021/np9006124 20038125 PMC2846203

[17] Carroll FI Ma W Yokota Y Lee JR Brieaddy LE Navarro HA Synthesis, nicotinic acetylcholine receptor binding, and antinociceptive properties of 3'-substituted deschloroepibatidine analogues. Novel nicotinic antagonists. J Med Chem (2005) 48(4):1221–8. 10.1021/jm040160b 15715488

[18] Carroll FI Ware R Brieaddy LE Navarro HA Damaj MI Martin BR. Synthesis, nicotinic acetylcholine receptor binding, and antinociceptive properties of 2'-fluoro-3'-(substituted phenyl)deschloroepibatidine analogues. Novel nicotinic antagonist. J Med Chem (2004) 47(18):4588–94. 10.1021/jm040078g 15317468

[19] Corrie LW Stokes C Wilkerson JL Carroll FI McMahon LR Papke RL. Nicotinic acetylcholine receptor accessory subunits determine the activity profile of epibatidine derivatives. Mol Pharmacol (2020) 98(4):328–42. 10.1124/molpharm.120.000037 32690626 PMC7485586

[20] Picciotto MR Zoli M Rimondini R Léna C Marubio LM Pich EM Acetylcholine receptors containing the beta2 subunit are involved in the reinforcing properties of nicotine. Nature (1998) 8(391):173–7. 10.1038/34413 9428762

[21] Kamble SH Obeng S León F Restrepo LF King TI Berthold EC Pharmacokinetic and pharmacodynamic consequences of cytochrome P450 3A inhibition on mitragynine metabolism in rats. J Pharmacol Exp Ther (2023) 385(3):180–92. 10.1124/jpet.122.001525 37019472 PMC10201580

[22] Tallarida RJ. Drug synergism and dose-effect data analysis. New York: Chapman and Hall/CRC (2000). p. 264.

[23] Abdrakhmanova GR Damaj MI Carroll FI Martin BR. 2-Fluoro-3-(4-nitro-phenyl)deschloroepibatidine is a novel potent competitive antagonist of human neuronal alpha4beta2 nAChRs. Mol Pharmacol (2006) 69(6):1945–52. 10.1124/mol.105.021782 16505153

[24] Ondachi P Castro A Luetje CW Damaj MI Mascarella SW Navarro HA Synthesis and nicotinic acetylcholine receptor *in vitro* and *in vivo* pharmacological properties of 2'-fluoro-3'-(substituted phenyl)deschloroepibatidine analogues of 2'-fluoro-3'-(4-nitrophenyl)deschloroepibatidine. J Med Chem (2012) 55(14):6512–22. 10.1021/jm300575y 22742586 PMC3431023

[25] Rollema H Shrikhande A Ward KM Tingley FD Coe JW O'Neill BT Pre-clinical properties of the alpha4beta2 nicotinic acetylcholine receptor partial agonists varenicline, cytisine and dianicline translate to clinical efficacy for nicotine dependence. Br J Pharmacol (2010) 160(2):334–45. 10.1111/j.1476-5381.2010.00682.x 20331614 PMC2874855

[26] Cunningham CS McMahon LR. The effects of nicotine, varenicline, and cytisine on schedule-controlled responding in mice: differences in α4β2 nicotinic receptor activation. Eur J Pharmacol (2011) 654(1):47–52. 10.1016/j.ejphar.2010.12.003 21172344 PMC3061304

[27] Mihalak KB Carroll FI Luetje CW. Varenicline is a partial agonist at alpha4beta2 and a full agonist at alpha7 neuronal nicotinic receptors. Mol Pharmacol (2006) 70(3):801–5. 10.1124/mol.106.025130 16766716

[28] Bagdas D Alkhlaif Y Jackson A Carroll FI Ditre JW Damaj MI. New insights on the effects of varenicline on nicotine reward, withdrawal and hyperalgesia in mice. Neuropharmacology (2018) 138:72–9. 10.1016/j.neuropharm.2018.05.025 29860196 PMC6054891

[29] Stolerman IP Chamberlain S Bizarro L Fernandes C Schalkwyk L. The role of nicotinic receptor alpha 7 subunits in nicotine discrimination. Neuropharmacology (2004) 46(3):363–71. 10.1016/j.neuropharm.2003.10.002 14975691

[30] de Moura FB Wilkerson JL McMahon LR. Unexpected loss of sensitivity to the nicotinic acetylcholine receptor antagonist activity of mecamylamine and dihydro-β-erythroidine in nicotine-tolerant mice. Brain Behav (2020) 10(4):e01581. 10.1002/brb3.1581 32092237 PMC7177571

[31] Davis L Pollock LJ Stone T. Visceral pain. Surg Gynecol Obstet (1932) 5:418–26.

[32] Wewers ME Dhatt RK Snively TA Tejwani GA. The effect of chronic administration of nicotine on antinociception, opioid receptor binding and met-enkelphalin levels in rats. Brain Res (1999) 822(1-2):107–13. 10.1016/s0006-8993(99)01095-1 10082888

[33] Wonnacott S. Presynaptic nicotinic ACh receptors. Trends Neurosci (1997) 20(2):92–8. 10.1016/s0166-2236(96)10073-4 9023878

[34] Zhu PJ Chiappinelli VA. Nicotine modulates evoked GABAergic transmission in the brain. J Neurophysiol (1999) 82(6):3041–5. 10.1152/jn.1999.82.6.3041 10601439

[35] Traynor JR. Epibatidine and pain. Br J Anaesth (1998) 81(1):69–76. 10.1093/bja/81.1.69 9771274

[36] Decker MW Meyer MD Sullivan JP. The therapeutic potential of nicotinic acetylcholine receptor agonists for pain control. Expert Opin Investig Drugs (2001) 10(10):1819–30. 10.1517/13543784.10.10.1819 11772288

[37] Damaj MI. Influence of gender and sex hormones on nicotine acute pharmacological effects in mice. J Pharmacol Exp Ther (2001) 296:132–40. 11123373

[38] Gopalakrishnan M Monteggia LM Anderson DJ Arneric SP Sullivan JP Donnelly-Roberts D Stable expression, pharmacologic properties and regulation of the human neuronal nicotinic acetylcholine alpha 4 beta 2 receptor. J Pharmacol Exp Ther (1996) 276:289–97. 8558445

[39] Bonhaus DW Bley KR Broka CA Fontana DJ Leung E Lewis R Characterization of the electrophysiological, biochemical and behavioral actions of epibatidine. J Pharmacol Exp Ther (1995) 272:1199–203. 7891333

[40] Cippitelli A Wu J Gaiolini KA Daniela M Jennifer S Michelle G AT-1001: a high-affinity α3β4 nAChR ligand with novel nicotine-suppressive pharmacology. Br J Pharmacol (2015) 172(7):1834–45. 10.1111/bph.13034 25440006 PMC4376460

[41] Overstreet DH. Behavioral characteristics of rat lines selected for differential hypothermic responses to cholinergic or serotonergic agonists. Behav Genet (2002) 32(5):335–48. 10.1023/a:1020262205227 12405515

