# Supplementary material for: Evaluation of novel epibatidine analogs in the rat nicotine drug discrimination assay and in the rat chronic constriction injury neuropathic pain model
Source: Adv Drug Alcohol Res. 2023 Sep 11;3:11622. doi: 10.3389/adar.2023.11622 (PMC10880765; doi:10.3389/adar.2023.11622)
Supplement: Supplementary file 1 [file DataSheet1.docx]

**Supplemental Methods**

*Drug Discrimination.* Eight operant-conditioning chambers (Model ENV-203; Med Associates Inc., Fairfax, VT) measuring 25-cm long x 25-cm wide x 31-cm high per chamber were each enclosed within a sound-attenuating cubicle equipped with a fan for ventilation and white noise to mask extraneous sounds. On the front wall of each chamber were two retractable, 5-cm-long response levers, 5 cm from the midline and 9 cm above the grid floor. A downward displacement of each lever with 0.20 N force defined a response. Two amber light-emitting diodes (LEDs) as stimulus lights were in a row above levers (one LED/lever). A receptacle for the delivery of 45-mg sucrose pellets (Dustless Precision Pellets® 45 mg, Sucrose, Bio-Serv) via a pellet dispenser (Model ENV-203-20; Med Associates Inc., Fairfax, VT) was mounted on the midline of the front wall between the two levers and 2 cm above the floor. A house light was centrally mounted on the upper wall facing the retractable levers. Each operant conditioning chamber was connected to a Dell desktop computer (Intel® Core™ i7-7700 3.60 GHz processor, 16.0 GB of RAM, Microsoft® Windows 10) through an interface (MED-SYST-8, Med Associates Inc.). Med-PC software version V (Med Associates Inc., Fairfax, VT) controlled experimental events and provided a record of responses.

*Drug Discrimination Procedures: A) Lever-response shaping.* Each subject was placed in their assigned chamber daily up to 120 min at the same time each day during the light period seven days per week. After the session each subject was returned to their home cage and 30 min later was fed a ration of food to maintain body weight. Sessions started with the presentation of one of the retractable levers and the illumination of the LED above the presented lever. Each downward deflection of the lever turned off the LEDs and activated the pellet dispenser for 0.1 seconds [fixed-ratio (FR) 1 schedule] followed by a 0.1-second time-out period during which LEDs were turned off, the house light was illuminated, and responding had no scheduled consequences; the retractable levers remained presented during this time-out period. The right- versus left-assignment of lever presentation was switched daily (i.e. right–left–right–left). After 50 reinforcers per session were presented within 20 min for four consecutive sessions under a FR1 and FR3 schedule of reinforcement and for two consecutive sessions under the FR5 schedule of reinforcement, the response requirement was increased to FR10. After 50 reinforcers per session were presented within 20 min for two consecutive sessions under the FR10 schedule of reinforcement, sessions for drug discrimination training commenced. The chamber assignments remained the same for each subject throughout the study.

*B) Drug Discrimination Training.* Rats were placed into a group that received nicotine (0.32 mg/kg, s.c.., administered 30 min prior to sessions). Following an injection of either the training dose or vehicle, subjects were returned to their home cage, and then placed into their assigned chamber after the pretreatment interval. Training sessions started with the presentation of one of the levers and the illumination of the LED above the presented lever under the FR10-response schedule of reinforcement. Each downward deflection of the injection-appropriate lever turned off the LED and activated the pellet dispenser delivering the sucrose pellet for 0.1 seconds followed by a 0.1-second time-out period during which the LED was turned off, the house light was illuminated, and responding had no scheduled consequences; the presented lever remained presented during this time-out period. Training sessions ended after 20 min or delivery of 50 pellets, whichever occurred first. Assignments of the levers to either drug or vehicle (e.g., left lever paired with drug; right lever paired with vehicle) were counterbalanced among subjects and remained the same for that subject. The order of drug and vehicle training followed a double-alternation sequence (i.e. right–left–left–right). After training with these parameters for 12 sessions, both levers were presented. Test sessions commenced when the following criteria were met individually for at least four consecutive sessions under the FR10-response schedule of reinforcement: 1) A minimum of 80% of the total responses was correct; and 2) the total of incorrect responses made prior to delivery of the first reinforcer was less than ten. After the first test session, each subject repeatedly underwent test sessions each time when the test criteria were met for at least one drug- and at least one vehicle-appropriate responding under the FR10-response schedule of reinforcement.

*C) Testing.* Test sessions were identical to the training sessions, except ten responses on either lever resulted in delivery of food and doses of test compounds were administered. Dose-effect assessments of each training drug were obtained once at the beginning of the study and a second time after tests with all other drugs were completed.

*Supplemental Figures*





**Supplemental Figure 1. Discriminative stimulus (top row) and rate-decreasing (bottom row) effects in rats trained to discriminate 0.32 mg/kg nicotine from saline do not undergo tolerance.** A.) The first and redetermined nicotine dose-related nicotine (0.32 mg/kg) discriminative stimulus, and B.) rate-decreasing effects. Top panels show drug-appropriate responding on the ordinates and drug dose in mg/kg (log scale) on the abscissae. Dashed line represents the percent responding on the nicotine-paired lever required to meet full discriminative stimulus criteria. Bottom panels show response rate normalized to saline control on the ordinates as function of drug dose in mg/kg (log scale) on the abscissae. Data reflect mean ± SEM, n = 7-8 rats (one rat was lost due to attrition).

**

**

**Supplemental Figure 2. DHβE (1 mg/kg) does not produce antagonism of discriminative stimulus (top row) and rate-decreasing (bottom row) effects in rats trained to discriminate 0.32 mg/kg nicotine from saline.** A.) At a low dose DHβE failed to produce a rightward shift of the nicotine dose response function for the nicotine (0.32 mg/kg) discriminative stimulus, and B.) response rate effects. C.) DHβE does not alter varenicline dose response function for the nicotine discriminative stimulus and D.) response rate effects. Top panels show drug-appropriate responding on the ordinates and drug dose in mg/kg (log scale) on the abscissae. Dashed line represents the percent responding on the nicotine-paired lever required to meet full discriminative stimulus criteria. Bottom panels show response rate normalized to saline control on the ordinates as function of drug dose in mg/kg (log scale) on the abscissae. Data reflect mean ± SEM, n = 7-8 rats (one rat was lost to attrition).

Supplemental Table 1

|  | Alone | | 1 mg/kg DHβE Pretreatment | | |
| --- | --- | --- | --- | --- | --- |
|  | Statistical Analysis | ED_50_ (95% Confidence limits) mg/kg | Statistical Analysis | ED_50_ (95% Confidence limits) mg/kg | Potency Ratio (DHβE/Alone) |
| Nicotine (1^st^ determination) | F (7, 28) = 17.96 P < 0.0001 | 0.12 (0.09 - 0.17) | F (7, 28) = 26.56 P < 0.001 | 0.11 (0.09 - 0.14) | 0.91 (0.52 – 1.55) |
| Nicotine (2nd determination) | F (6, 24) = 20.30 | 0.19 (0.14 -0.29) | N.A. | N.A. | N.A. |
| Varenicline | F (7, 38) = 11.49 P < 0.001 | 0.30 (0.21-0.42) | F (6, 36) = 14.60 P < 0.001 | 0.37 (0.31- 0.45) | 1.23 (0.73 – 2.14) |

Statistics Related to Nicotine drug discrimination dose effect determinations and 1 mg/kg DHβE pretreatment
